# Genomic characterization of SARS-CoV-2 from vaccine breakthrough cases in Allegheny County, Pennsylvania

**DOI:** 10.1371/journal.pone.0272954

**Published:** 2022-08-31

**Authors:** Kady D. Waggle, Marissa P. Griffith, Lei Zhu, Vaughn S. Cooper, Daniel J. Snyder, Vatsala Srinivasa, Tung Phan, Alan Wells, Graham M. Snyder, Daria Van Tyne, Lee H. Harrison, Jane W. Marsh

**Affiliations:** 1 Microbial Genomics Epidemiology Laboratory, Center for Genomic Epidemiology, University of Pittsburgh, Pittsburgh, Pennsylvania, United States of America; 2 Division of Infectious Diseases, University of Pittsburgh School of Medicine, Pittsburgh, Pennsylvania, United States of America; 3 Department of Microbiology and Molecular Genetics and Center for Evolutionary Biology and Medicine, University of Pittsburgh School of Medicine, Pittsburgh, Pennsylvania, United States of America; 4 Microbial Genome Sequencing Center, LLC, Pittsburgh, Pennsylvania, United States of America; 5 Department of Pathology, University of Pittsburgh, Pittsburgh, Pennsylvania, United States of America; 6 Department of Infection Control and Hospital Epidemiology, UPMC Presbyterian, Pittsburgh, Pennsylvania, United States of America; 7 Department of Epidemiology, Graduate School of Public Health, University of Pittsburgh, Pittsburgh, Pennsylvania, United States of America; Instituto de Salud Carlos III, SPAIN

## Abstract

We performed whole genome sequencing on SARS-CoV-2 from 59 vaccinated individuals from southwest Pennsylvania who tested positive between February and September, 2021. A comparison of mutations among vaccine breakthrough cases to a time-matched control group identified potential adaptive responses of SARS-CoV-2 to vaccination.

## Introduction

SARS-CoV-2 vaccination prevents severe disease and hospitalizations and is highly effective at reducing COVID-19 mortality [1,2]. SARS-CoV-2 evolution may diminish vaccine effectiveness. Vaccine breakthrough may be due to waning immunity and/or viral immune escape [3,4].

A Variant of Concern (VOC) is defined by genetic changes associated with increased SARS-CoV-2 transmissibility, virulence and/or decreased effectiveness of public health strategies, including vaccination (www.who.int/en/activities/tracking-SARS-CoV-2-variants). As the Delta VOC spread in 2021 a reduction in vaccine-induced neutralizing antibodies was observed [5]. The emergence of the Omicron VOC recently has raised concerns of potential escape from vaccine-induced immunity [6].

Whole genome sequencing (WGS) of SARS-CoV-2 vaccine breakthrough cases can reveal mutations that may contribute to viral immune escape. In this study, we performed WGS on 59 SARS-CoV-2 positive samples collected from vaccinated individuals in Allegheny County, Pennsylvania to examine viral evolution in response to vaccination.

## Materials and methods

A convenience sample of residual SARS-CoV-2-positive nasopharyngeal swabs from 55 healthcare workers and 4 inpatient hospital-acquired infections (HAIs) were collected between February 1, 2021 and September 12, 2021 (S1 Table). HAIs were defined as inpatients who developed COVID-19 ≥3 days post-admission. A vaccine breakthrough case was defined as a SARS-CoV-2-positive test from an individual two weeks or more after two-dose mRNA vaccination. Samples with cycle threshold (Ct) values less than 33 were identified using either the CDC 2019-nCOV RT-PCR Diagnostic Panel or the Cepheid Xpert Xpress SARS-CoV-2 Test. Total RNA was extracted using the QiaAmp Viral RNA Mini kit [Qiagen] and WGS was performed using the ARTIC v3 protocol [7]. Sequencing libraries generated using Illumina Nextera chemistry were sequenced on a NextSeq 2000 high-output flowcell. ARTIC primers were trimmed using iVar v1.3.1 [8]. Trimmed reads were aligned to the Wuhan reference genome (MN908947) and single nucleotide polymorphisms (SNPs) were identified using BreSeq v0.33.2 with default parameters [9]. Only genomes with less than 5% ambiguous nucleotides were included. Genetic lineages were identified using the Phylogenetic Assignment of Named Global Outbreak LINeages (Pangolin) software v3.16.1 [10], with pangoLEARN version 2021-11-18. Multiple sequence alignment to MN908947 was performed with MAFFT v7.475 [11], and a time-scaled phylogenetic tree was generated using TreeTime [12] and visualized using the ggtree package in R. The frequency of SNPs resulting in non-synonymous mutations in viral coding regions was examined. Frequencies of enriched non-synonymous mutations occurring in >2 but <16 (Alpha) and <37 (Delta) genomes were used to eliminate low frequency (<2) and lineage specific SNPs. Bivariate analyses were conducted using Chi-square or Fisher’s exact tests to detect differences of non-synonymous mutations between vaccine breakthrough and GISAID EpiFlu™ control genomes collected from Pennsylvania during the same time period as vaccine breakthrough collection. Genomes that clustered with ≤2 SNPs were excluded from the analysis to remove bias from potential transmission clusters. Associations between non-synonymous mutations and vaccine breakthrough cases were assessed using logistic regression adjusted for sample collection time. Odds ratio (OR) and corresponding 95% confidence interval (95% CI) were determined for each significant association. Statistical analyses were conducted using SAS version 9.4 (SAS Institute Inc., Cary, NC, USA). Two-sided p-values ≤0.05 were considered statistically significant. This study was approved by the University of Pittsburgh Internal Review Board (Protocol:22010033).

## Results and discussion

Among 59 vaccine breakthrough cases, 49 (83%) received Pfizer-BioNTech and 6 (10%) received Moderna; vaccine type was unavailable for 4 (7%) cases. All cases presented with mild respiratory viral symptoms, headache, body aches, and/or fever (S1 Table). Four cases were hospital-acquired infections (7%). The median number of days from last vaccine dose to symptom onset was 183 days (IQR: 94–209) among 54 cases with available data; median number of days to symptom onset among 44 Pfizer-BioNTech and 6 Moderna recipients with available data was 142 (IQR: 79–217) and 193 (IQR: 102–208) days respectively, with no significant difference by vaccine manufacturer (p = 0.917). Median age of 57 cases with available birth dates was 37 years (IQR: 29–45), and 93% of cases (55/59) were healthcare workers. The average PCR Ct value from 57 samples with available data was 20 (Median: 20; IQR: 17–24).

**Fig 1:**
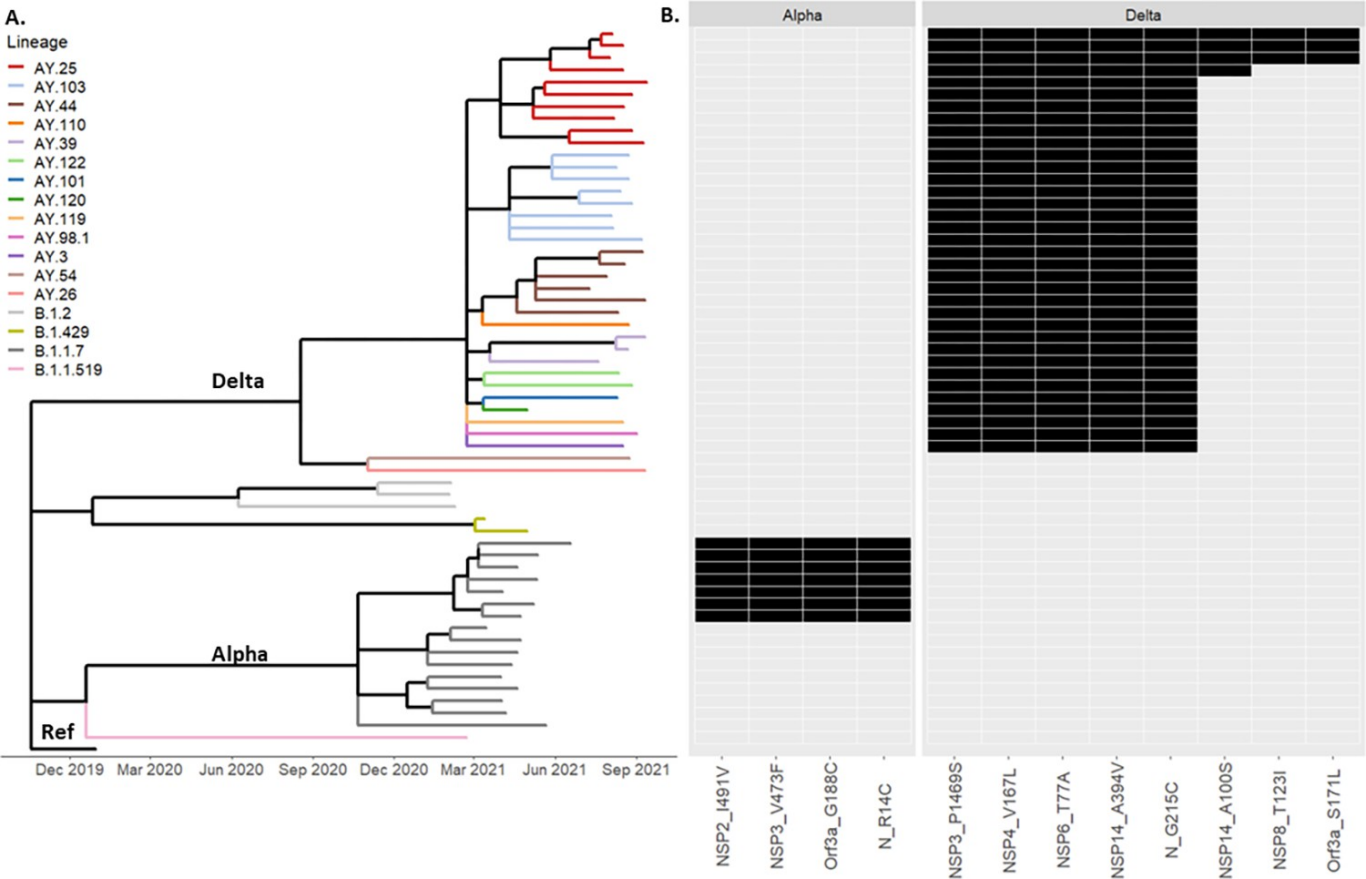
Lineages and mutations among SARS-CoV-2 vaccine breakthrough cases. (A) Time-scaled phylogeny of 59 SARS-CoV-2 genomes collected from vaccine breakthrough cases in Allegheny County, PA; Ref. Wuhan reference genome (MN908947). (B) Significantly enriched non-synonymous mutations among Alpha and Delta VOCs; black, present; white, absent.

Five major Pangolin lineages were identified by WGS: B.1.1.7 (Alpha) n = 16, B.1.1.519 n = 1, B.1.2 n = 3, B.1.429 n = 2 and Delta n = 37 (Figs 1A and S1). A time-scaled phylogeny showed samples belonging to lineages B.1.2, B.1.1.519 and B.1.429 collected in February and March 2021, followed by emergence of Alpha (April–July) and Delta (May–September) VOCs (Fig 1A). Thirty-seven genomes belonging to the Delta VOC comprised thirteen AY sublineages (S1 Table). Three major AY sublineages were detected: AY.25 (n = 10), AY.44 (n = 6) and AY.103 (n = 8).

Among the 16 Alpha VOC vaccine breakthrough genomes, 4 non-synonymous mutations were significantly enriched (p < .0001) relative to Pennsylvania (PA) Alpha VOC genomes collected from the same time period (Fig 1, S2 Table). These four mutations (NSP2_I491V, NSP3_V473F, Orf3a_G188C and N_R14C) defined an Alpha sublineage present in 44% of vaccine breakthrough genomes and only 6% of control genomes (p < .0001).

Among the 37 Delta VOC breakthrough genomes, 8 non-synonymous mutations were significantly enriched relative to control genomes (Fig 1, S3 Table); no mutations were shared with Alpha vaccine breakthrough genomes. Five mutations defined a major branch of the Delta phylogeny (Fig 1B) and occurred in 95% of vaccine breakthrough and 81% of control genomes (p = 0.034, S3 Table). The majority of these mutations (4/5, 80%) occurred in genes encoding non-structural proteins (NSP3, NSP4, NSP6 and NSP14). Three mutations were enriched in a subset of AY25 Delta vaccine breakthrough genomes relative to control genomes (Fisher’s exact, p < .0001): NSP8_T123I (8% vs. 0), NSP14_A100S (11% vs. 1%) and Orf3A_S171L (8% vs. 0). Together, these data demonstrate accumulation and significant enrichment of non-synonymous mutations among vaccine breakthrough cases in Allegheny County relative to statewide control cases.

In this study, genomic characterization of 59 SARS-CoV-2 vaccine breakthrough cases was performed to investigate SARS-CoV-2 evolution in a vaccinated population. Vaccine-induced immunity might lead to mutations in the Spike protein, the target of many vaccines. While enrichment for non-synonymous mutations in Spike was not observed among vaccine breakthrough genomes in this study, multiple substitutions in genes encoding non-structural and accessory proteins were enriched among vaccine breakthrough genomes compared to control genomes. Mutations in SARS-CoV-2 non-structural proteins associated with viral replication may provide fitness advantages. A recent study suggested that multiple non-structural protein mutations among vaccine breakthrough genomes may contribute to emergence and persistence of a Delta subvariant [13].

In general, the diversity of vaccine breakthrough genomes observed in this study were consistent with the occurrence of major SARS-CoV-2 lineages circulating in the US during the spring and summer of 2021 [14,15]. Higher viral loads and increased odds of vaccine breakthrough among patients infected with Delta versus Alpha have been reported [16,17]. These observations may be due to a combination of waning host immunity and viral evolution. In Israel, rates of SARS-CoV-2 infection increased as time from vaccination increased in different age groups [18]. In our study, approximately 50% of vaccine breakthrough cases (30/59) occurred in individuals who received vaccinations ≥5 months prior to symptom onset.

Limitations of our study include a small sample size and use of state-wide control genomes (without knowledge of vaccination status) for a county-level investigation. In addition, we do not know whether HCWs in this convenience sample are representative of all HCWs with breakthrough infection. Larger WGS surveillance studies are necessary to track the evolution of SARS-CoV-2 in vaccinated populations, and identify potential immune escape variants.

## Conclusion

In conclusion, SARS-CoV-2 vaccine breakthrough cases observed in our study are likely the result of a combination of waning immunity, the prevalent SARS-CoV-2 VOCs circulating at the time of the study, and viral evolution.

## Supporting information

S1 Fig Diversity of SARS-CoV-2 genomes from 59 vaccine breakthrough cases. (Fig A in S1 Fig) Maximum-likelihood phylogeny of 59 SARS-CoV-2 genomes collected from vaccine breakthrough cases in Allegheny County, PA; Ref. Wuhan reference genome (MN908947). Branches with bootstrap confidence values >90 are indicated; scale = 1 x 10^−4^ nucleotide substitutions per site (Fig B in S1 Fig) Significantly enriched non-synonymous mutations among Alpha and Delta VOCs; black, present; white, absent.
(TIF)

S1 Table Demographics of 59 vaccine breakthrough cases. (DOCX)

S2 Table Non-synonymous mutations enriched among Alpha VOC in vaccine breakthrough (Vax Bt) cases (n = 16) relative to Pennsylvania (PA) control cases (n = 2,466). (DOCX)

S3 Table Non-synonymous mutations enriched among Delta VOC in vaccine breakthrough (Vax Bt) cases (n = 37) relative to Pennsylvania (PA) control cases (n = 2,273). (DOCX)

## References

[1] Chia PY, Xiang Ong SW, Chiew CJ, Ang LW, Chavatte JM, Mak TM, et al. Virological and serological kinetics of SARS-CoV-2 Delta variant vaccine-breakthrough infections: a multi-center cohort study. Clin Microbiol Infect. 2021. 10.1016/j.cmi.2021.11.010 PMC8608661 34826623

[2] Tregoning JS, Flight KE, Higham SL, Wang Z, Pierce BF. Progress of the COVID-19 vaccine effort: viruses, vaccines and variants versus efficacy, effectiveness and escape. Nat Rev Immunol. 2021;21(10):626–36. doi: 10.1038/s41577-021-00592-1 34373623 PMC8351583

[3] Duerr R, Dimartino D, Marier C, Zappile P, Wang G, Lighter J, et al. Dominance of Alpha and Iota variants in SARS-CoV-2 vaccine breakthrough infections in New York City. J Clin Invest. 2021;131(18). doi: 10.1172/JCI152702 34375308 PMC8439605

[4] Pollett SD, Richard SA, Fries AC, Simons MP, Mende K, Lalani T, et al. The SARS-CoV-2 mRNA vaccine breakthrough infection phenotype includes significant symptoms, live virus shedding, and viral genetic diversity. Clin Infect Dis. 2021. 10.1093/cid/ciab543 PMC8906702 34117878

[5] Mlcochova P, Kemp SA, Dhar MS, Papa G, Meng B, Ferreira I, et al. SARS-CoV-2 B.1.617.2 Delta variant replication and immune evasion. Nature. 2021;599(7883):114–9. doi: 10.1038/s41586-021-03944-y 34488225 PMC8566220

[6] Dolgin E. Omicron is supercharging the COVID vaccine booster debate. Nature. 2021. doi: 10.1038/d41586-021-03592-2 34862505

[7] Tyson JR, James P, Stoddart D, Sparks N, Wickenhagen A, Hall G, et al. Improvements to the ARTIC multiplex PCR method for SARS-CoV-2 genome sequencing using nanopore. bioRxiv. 2020.

[8] Grubaugh ND, Gangavarapu K, Quick J, Matteson NL, De Jesus JG, Main BJ, et al. An amplicon-based sequencing framework for accurately measuring intrahost virus diversity using PrimalSeq and iVar. Genome Biol. 2019;20(1):8. doi: 10.1186/s13059-018-1618-7 30621750 PMC6325816

[9] Deatherage DE, Traverse CC, Wolf LN, Barrick JE. Detecting rare structural variation in evolving microbial populations from new sequence junctions using breseq. Front Genet. 2014;5:468. doi: 10.3389/fgene.2014.00468 25653667 PMC4301190

[10] O’Toole A, Scher E, Underwood A, Jackson B, Hill V, McCrone JT, et al. Assignment of epidemiological lineages in an emerging pandemic using the pangolin tool. Virus Evol. 2021;7(2):veab064. doi: 10.1093/ve/veab064 34527285 PMC8344591

[11] Katoh K, Standley DM. MAFFT multiple sequence alignment software version 7: improvements in performance and usability. Mol Biol Evol. 2013;30(4):772–80. doi: 10.1093/molbev/mst010 23329690 PMC3603318

[12] Sagulenko P, Puller V, Neher RA. TreeTime: Maximum-likelihood phylodynamic analysis. Virus Evol. 2018;4(1):vex042. doi: 10.1093/ve/vex042 29340210 PMC5758920

[13] Brinkac L, Diepold S, Mitchell S, Sarnese S, Kolakowski LF, Nelson WM, et al. SARS-CoV-2 Delta variant isolates from vaccinated individuals. BMC Genomics. 2022;23(1):417. doi: 10.1186/s12864-022-08652-z 35658876 PMC9166184

[14] Malden DE, Bruxvoort KJ, Tseng HF, Ackerson B, Choi SK, Florea A, et al. Distribution of SARS-CoV-2 Variants in a Large Integrated Health Care System—California, March-July 2021. MMWR Morb Mortal Wkly Rep. 2021;70(40):1415–9. doi: 10.15585/mmwr.mm7040a4 34618801 PMC8519275

[15] Taylor CA, Patel K, Pham H, Whitaker M, Anglin O, Kambhampati AK, et al. Severity of Disease Among Adults Hospitalized with Laboratory-Confirmed COVID-19 Before and During the Period of SARS-CoV-2 B.1.617.2 (Delta) Predominance—COVID-NET, 14 States, January-August 2021. MMWR Morb Mortal Wkly Rep. 2021;70(43):1513–9. doi: 10.15585/mmwr.mm7043e1 34710076 PMC8553023

[16] Christensen PA, Olsen RJ, Long SW, Subedi S, Davis JJ, Hodjat P, et al. Delta Variants of SARS-CoV-2 Cause Significantly Increased Vaccine Breakthrough COVID-19 Cases in Houston, Texas. Am J Pathol. 2021. doi: 10.1016/j.ajpath.2021.10.019 34774517 PMC8580569

[17] Kislaya I, Rodrigues EF, Borges V, Gomes JP, Sousa C, Almeida JP, et al. Comparative Effectiveness of Coronavirus Vaccine in Preventing Breakthrough Infections among Vaccinated Persons Infected with Delta and Alpha Variants. Emerg Infect Dis. 2021;28(2). doi: 10.3201/eid2802.211789 34876242 PMC8798697

[18] Goldberg Y, Mandel M, Bar-On YM, Bodenheimer O, Freedman L, Haas EJ, et al. Waning Immunity after the BNT162b2 Vaccine in Israel. N Engl J Med. 2021;385(24):e85. doi: 10.1056/NEJMoa2114228 34706170 PMC8609604

