# Supplementary figures and images for: Genomic characterization of SARS-CoV-2 from vaccine breakthrough cases in Allegheny County, Pennsylvania
Source: PLoS One. 2022 Aug 31;17(8):e0272954. doi: 10.1371/journal.pone.0272954 (PMC9432771; doi:10.1371/journal.pone.0272954)

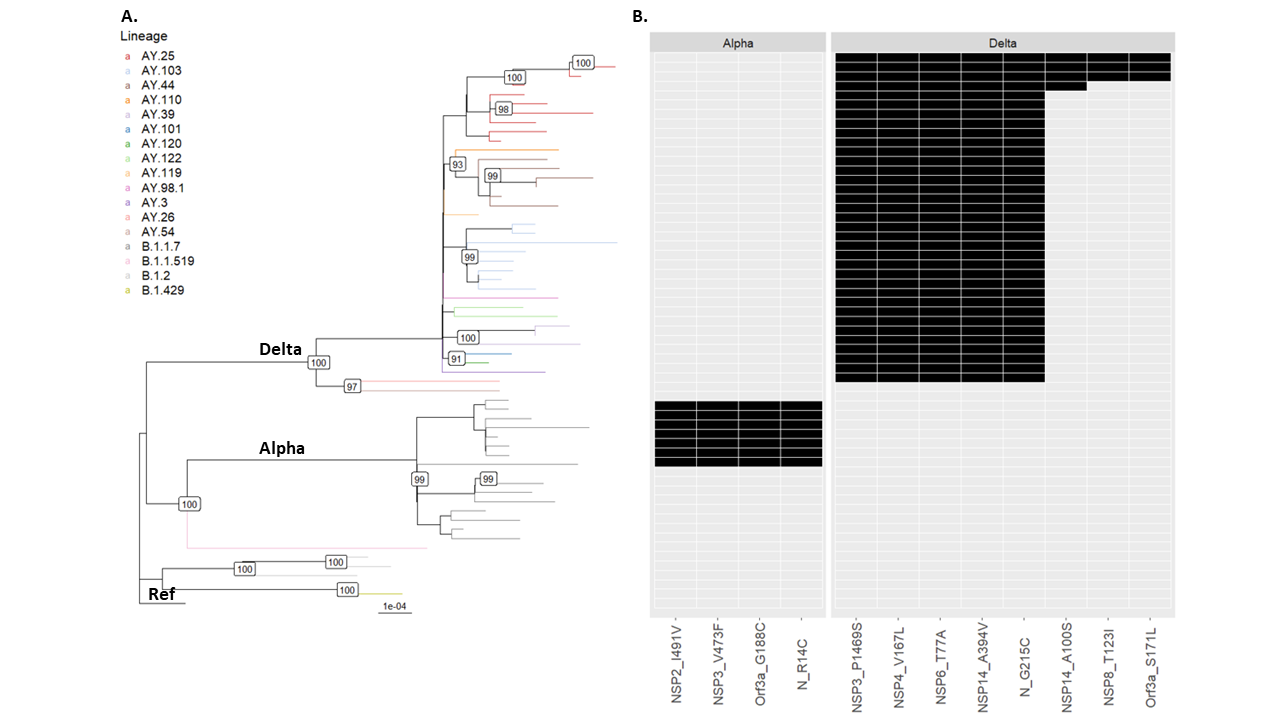

Supplement: S1 Fig — (Fig A in S1 Fig) Maximum-likelihood phylogeny of 59 SARS-CoV-2 genomes collected from vaccine breakthrough cases in Allegheny County, PA; Ref. Wuhan reference genome (MN908947). Branches with bootstrap confidence values >90 are indicated; scale = 1 x 10^−4^ nucleotide substitutions per site (Fig B in S1 Fig) Significantly enriched non-synonymous mutations among Alpha and Delta VOCs; black, present; white, absent. (TIF) [file pone.0272954.s001.tif]
