# Supplementary material for: Genomic characterization of SARS-CoV-2 from vaccine breakthrough cases in Allegheny County, Pennsylvania
Source: PLoS One. 2022 Aug 31;17(8):e0272954. doi: 10.1371/journal.pone.0272954 (PMC9432771; doi:10.1371/journal.pone.0272954)
Supplement: S1 Table — (DOCX) [file pone.0272954.s002.docx]

| S1Table: Demographics of 59 vaccine breakthrough cases. | | | | | | | | |  | | |  |
| --- | --- | --- | --- | --- | --- | --- | --- | --- | --- | --- | --- | --- |
| Sample Number | Vaccine | Ct | Lineage | Hospitalized | Symptomatic | Days to symptom onset | HCW | Symptoms | | Age | GISAID ID | |
| 1 | Pfizer | 18 | B.1.429 | NO | YES | 50 | YES | congestion, sore throat | | 34 | EPI_ISL_1373684 | |
| 2 | Pfizer | 22 | B.1.429 | NO | YES | 108 | YES | dry cough, sore throat, nasal congestion, headache, body aches, fatigue | | 49 | EPI_ISL_2775813 | |
| 3 | Pfizer | 27 | B.1.2 | NO | YES | 25 | YES | nasal congestion, scratchy throat, mild cough | | 26 | EPI_ISL_1137185 | |
| 4 | Pfizer | 21 | B.1.2 | NO | YES | 25 | YES | congestion, loss taste/smell | | 46 | EPI_ISL_1137180 | |
| 5 | Pfizer | 26 | B.1.2 | NO | YES | 24 | YES | congestion, headache, diarrhea | | 37 | EPI_ISL_1137176 | |
| 6 | Pfizer | 17 | B.1.1.7 | NO | YES | na | YES | mild respiratory symptoms | | na | EPI_ISL_1373685 | |
| 7 | Pfizer | 22 | B.1.1.7 | NO | YES | 75 | YES | shortness of breath with cough | | 33 | EPI_ISL_1732422 | |
| 8 | Moderna | 21 | B.1.1.7 | NO | YES | 63 | YES | nasal congestion, sore throat, headache | | 33 | EPI_ISL_1732423 | |
| 9 | Pfizer | 15 | B.1.1.7 | NO | YES | na | YES | mild upper respiratory symptoms, cough | | na | EPI_ISL_1732495 | |
| 10 | Pfizer | 15 | B.1.1.7 | NO | YES | 81 | YES | unknown | | 50 | EPI_ISL_1732502 | |
| 11 | Pfizer | 14 | B.1.1.7 | NO | YES | 94 | YES | congestion, fever, myalgia, headache | | 36 | EPI_ISL_1732503 | |
| 12 | Pfizer | 27 | B.1.1.7 | NO | YES | 96 | YES | fatigue, congestion, scratchy throat | | 27 | EPI_ISL_2775797 | |
| 13 | Pfizer | 24 | B.1.1.7 | NO | YES | 60 | YES | sinus congestion/pressure/ frontal headache, allergy like symptoms, loss taste/smell | | 46 | EPI_ISL_2775798 | |
| 14 | Pfizer | 22 | B.1.1.7 | NO | YES | 72 | YES | congestion, headache, loss taste/smell, slight cough, fatigue | | 32 | EPI_ISL_2775799 | |
| 15 | Pfizer | 18 | B.1.1.7 | NO | YES | 77 | YES | congestion, sinus pressure | | 33 | EPI_ISL_2775811 | |
| 16 | Pfizer | 20 | B.1.1.7 | NO | YES | 99 | YES | runny nose, congestion | | 31 | EPI_ISL_2775812 | |
| 17 | Pfizer | 19 | B.1.1.7 | NO | YES | 103 | YES | low grade fever, cough, headache, runny nose, body aches, chills | | 30 | EPI_ISL_2775863 | |
| 18 | Moderna | 27 | B.1.1.7 | NO | YES | 102 | YES | vomiting, shortness of breath, cough, loss of taste | | 38 | EPI_ISL_2775864 | |
| 19 | Pfizer | 19 | B.1.1.7 | NO | YES | 124 | YES | headache, nasal congestion, body aches | | 45 | EPI_ISL_2775865 | |
| 20 | Pfizer | 22 | B.1.1.7 | NO | YES | 135 | YES | nasal congestion, head cold | | 49 | EPI_ISL_2775866 | |
| 21 | Pfizer | 26 | B.1.1.7 | NO | YES | 112 | YES | headache, fatigue, loss of taste/smell | | 46 | EPI_ISL_3322815 | |
| 22 | Pfizer | 25 | B.1.1.519 | NO | YES | 30 | YES | stuffy nose and loss of smell | | 36 | EPI_ISL_1373724 | |
| 23 | Pfizer | 19 | AY.120 | NO | YES | 106 | YES | cough, body aches, runny nose, fever | | 32 | EPI_ISL_5327540 | |
| 24 | Pfizer | na | AY.44 | NO | YES | 162 | YES | fever just broke, nasal congestion, runny nose | | 35 | EPI_ISL_3322804 | |
| 25 | Pfizer | na | AY.39 | NO | YES | 149 | YES | fever, chills, body aches, cough | | 44 | EPI_ISL_3341934 | |
| 26 | Pfizer | 15 | AY.44 | NO | YES | 199 | YES | cough, sinus pressure, congestion, fever | | 27 | EPI_ISL_5327541 | |
| 27 | na | 21 | AY.103 | NO | YES | 197 | YES | hoarse voice, fever, pneumonia on x-ray | | 53 | EPI_ISL_5327542 | |
| 28 | na | 14 | AY.101 | NO | YES | 211 | YES | fever, loss of taste and smell, fatigue, body aches | | 42 | EPI_ISL_5327538 | |
| 29 | na | 18 | AY.44 | NO | YES | 186 | YES | fatigue, sore throat, headache, chills, fever | | 29 | EPI_ISL_5327593 | |
| 30 | Pfizer | 24 | AY.122 | NO | YES | 196 | YES | cough, nasal congestion, sore throat, body aches | | 38 | EPI_ISL_5327553 | |
| 31 | Pfizer | 18 | AY.103 | YES | YES | na | NO | unknown | | 59 | EPI_ISL_5327594 | |
| 32 | Pfizer | 22 | AY.39 | NO | YES | 237 | YES | congestion, body aches, headache, mild cough | | 26 | EPI_ISL_5327595 | |
| 33 | Pfizer | 27 | AY.103 | NO | YES | 182 | YES | loss taste/smell, stuffy nose | | 38 | EPI_ISL_5327541 | |
| 34 | na | 27 | AY.103 | NO | YES | 183 | YES | phlegm, sense of smell slightly decreased | | 28 | EPI_ISL_5327590 | |
| 35 | Moderna | 20 | AY.119 | NO | YES | 200 | YES | sore throat, headache, fatigue, no taste, congestion, sinus pressure | | 45 | EPI_ISL_5327583 | |
| 36 | Pfizer | 19 | AY.44 | NO | YES | 209 | YES | fever/chills | | 27 | EPI_ISL_5327560 | |
| 37 | Pfizer | 24 | AY.103 | NO | YES | 67 | YES | cough, congestion, shortness of breath, sore throat, body aches | | 42 | EPI_ISL_5327561 | |
| 38 | Moderna | 14 | AY.110 | NO | YES | 208 | YES | congestion, runny nose | | 32 | EPI_ISL_5327584 | |
| 39 | Pfizer | 14 | AY.103 | NO | YES | 225 | YES | nasal congestion, sneezing, runny nose, cough, fever | | 31 | EPI_ISL_5327562 | |
| 40 | Pfizer | 16 | AY.39 | NO | YES | 220 | YES | congestion, body aches, sore throat, chills, shortness of breath | | 46 | EPI_ISL_5327563 | |
| 41 | Pfizer | 16 | AY.103 | NO | YES | 229 | YES | fever, cough, headache, body aches, congestion | | 40 | EPI_ISL_5327567 | |
| 42 | Pfizer | 15 | AY.122 | NO | YES | 207 | YES | dry cough, fatigue, mild shortness of breath, headache, fever | | 68 | EPI_ISL_5327588 | |
| 43 | Pfizer | 21 | AY.98.1 | NO | YES | 103 | YES | sore throat, headache, runny nose | | 26 | EPI_ISL_5327568 | |
| 44 | Pfizer | 26 | AY.103 | NO | YES | 223 | YES | body aches, rigors, chills, cough, sore throat, headache | | 53 | EPI_ISL_5327569 | |
| 45 | Pfizer | 25 | AY.44 | NO | YES | 238 | YES | headache, cough, body aches, fever, congestion | | 27 | EPI_ISL_5327570 | |
| 46 | Pfizer | 22 | AY.44 | NO | YES | 244 | YES | dry cough | | 38 | EPI_ISL_5327573 | |
| 47 | Pfizer | 15 | AY.3 | NO | YES | 217 | YES | headache, sore throat, congestion, cough, fever | | 27 | EPI_ISL_5327555 | |
| 48 | Pfizer | 24 | AY.26 | NO | YES | 46 | YES | runny nose, congestion, sore throat, headache | | 32 | EPI_ISL_5327574 | |
| 49 | Pfizer | 19 | AY.25 | NO | YES | 202 | YES | fever, cough, sore throat | | 37 | EPI_ISL_5327589 | |
| 50 | Pfizer | 18 | AY.25 | YES | YES | na | NO | na | | 65 | EPI_ISL_5327549 | |
| 51 | Pfizer | 15 | AY.25 | YES | YES | na | NO | na | | 79 | EPI_ISL_5327552 | |
| 52 | Moderna | 15 | AY.25 | NO | YES | 186 | YES | body aches, fever, chills, sinus pressure, sore throat | | 27 | EPI_ISL_5327554 | |
| 53 | Pfizer | 20 | AY.25 | NO | YES | 207 | YES | cough, body aches, headache, low grade temp, congestion | | 28 | EPI_ISL_5327556 | |
| 54 | Pfizer | 17 | AY.25 | NO | YES | 216 | YES | fever, body aches, headache | | 39 | EPI_ISL_5327557 | |
| 55 | Pfizer | 19 | AY.25 | NO | YES | 229 | YES | congestion, post nasal drip, cough | | 27 | EPI_ISL_5327566 | |
| 56 | Moderna | 27 | AY.25 | NO | YES | 209 | YES | dry cough | | 25 | EPI_ISL_5327586 | |
| 57 | Pfizer | 23 | AY.25 | NO | YES | 232 | YES | cough, headache | | 28 | EPI_ISL_5327571 | |
| 58 | Pfizer | 20 | AY.25 | NO | YES | 241 | YES | congestion, vomiting | | 26 | EPI_ISL_5327575 | |
| 59 | Pfizer | 17 | AY.54 | NO | YES | 208 | YES | runny nose, sore throat, headache | | 38 | EPI_ISL_5327587 | |

Ct, PCR cycle threshold; na, not available
