# Supplementary material for: Genomic characterization of SARS-CoV-2 from vaccine breakthrough cases in Allegheny County, Pennsylvania
Source: PLoS One. 2022 Aug 31;17(8):e0272954. doi: 10.1371/journal.pone.0272954 (PMC9432771; doi:10.1371/journal.pone.0272954)
Supplement: S2 Table — (DOCX) [file pone.0272954.s003.docx]

S2 Table. Non-synonymous mutations enriched among Alpha VOC in vaccine breakthrough (Vax Bt) cases (n=16) relative to Pennsylvania (PA) control cases (n=2,466)

|  | Vax Bt  N (%) | PA  N (%) | p-value^ | Odds*  Ratio | 95% CI |
| --- | --- | --- | --- | --- | --- |
| NSP2_I491V | 7 (44) | 150 (6) | <.0001 | 11.64 | 4.27 - 31.75 |
| NSP3_V473F | 7 (44) | 150 (6) | <.0001 | 11.64 | 4.27 - 31.75 |
| Orf3A_G188C | 7 (44) | 151 (6) | <.0001 | 11.56 | 4.24 - 31.53 |
| N_R14C | 7 (44) | 141 (6) | <.0001 | 12.40 | 4.54 - 33.88 |

NSP, non-structural protein; N, nucleocapsid;

^ Chi-square test p values;

* Adjusted Odds ratios: model adjusted for collect date.
