# Supplementary material for: Genomic characterization of SARS-CoV-2 from vaccine breakthrough cases in Allegheny County, Pennsylvania
Source: PLoS One. 2022 Aug 31;17(8):e0272954. doi: 10.1371/journal.pone.0272954 (PMC9432771; doi:10.1371/journal.pone.0272954)
Supplement: S3 Table — (DOCX) [file pone.0272954.s004.docx]

S3 Table. Non-synonymous mutations enriched among Delta VOC in vaccine breakthrough (Vax Bt) cases (n=37) relative to Pennsylvania (PA) control cases (n=2,273)

|  | Vax Bt  N (%) | PA  N (%) | p-value^ | Odds Ratio* | 95% CI |
| --- | --- | --- | --- | --- | --- |
| NSP3_P1469S | 35 (95) | 1832 (81) | 0.033 | 4.09 | 0.97 - 17.18 |
| NSP4_V167L | 35 (95) | 1848 (81) | 0.034 | 3.90 | 0.93 - 16.40 |
| NSP6_T77A | 35 (95) | 1848 (81) | 0.034 | 3.90 | 0.93 - 16.40 |
| NSP8_T123I | 3 (8) | 4 (0) | <.001 | 54.89 | 11.65 - 258.66 |
| NSP14_A100S | 4 (11) | 15 (1) | <.001 | 18.32 | 5.77 - 58.22 |
| NSP14_A394V | 35 (95) | 1849 (81) | 0.018 | 3.89 | 0.93 - 16.35 |
| Orf3a_S171L | 3 (8) | 10 (0) | 0.001 | 21.31 | 5.55 - 81.82 |
| N_G215C | 35 (95) | 1842 (81) | 0.033 | 3.97 | 0.95 - 16.69 |

NSP, non-structural protein; N, nucleocapsid

^ Fisher’s exact test p values;

* Adjusted Odds ratios: model adjusted for collect date.
